# Influence of the cultivation model on the posture and back pain prevalence of strawberry producers

**DOI:** 10.47626/1679-4435-2022-803

**Published:** 2023-02-13

**Authors:** Jackson de Souza, Tuane Miorelli Rigatti, William Dhein, Marcelo La Torre

**Affiliations:** 1 Curso de Fisioterapia, Universidade do Vale do Rio dos Sinos, São Leopoldo, RS, Brazil; 2 Curso de Fisioterapia, Centro Universitário da Serra Gaúcha, Caxias do Sul, RS, Brazil

**Keywords:** agriculture, spine, pain, agricultura, coluna vertebral, dor

## Abstract

**Introduction:**

Strawberry cultivation is an important source of income for Brazilian farmers. Cultivation can be done using the traditional model, in which producers must flex their trunk to handle seedlings, or using the hydroponic model, which involves an upright posture.

**Objectives:**

To verify the influence of cultivation model on posture and back pain prevalence among strawberry producers.

**Methods:**

A total of 26 strawberry producers who used traditional or hydroponic methods participated in the study. The angular values of the curvatures of the thoracic and lumbar spine in the sagittal plane were obtained using the “flexicurve” method, while pain prevalence was determined with Souza & Krieger’s back pain questionnaire. The *t*-test for independent samples and the chi-square test were used to compare group results.

**Results:**

Growers using the traditional model had greater thoracic spine curvature (45.5 [SD, 26.2 ]) than those who used the hydroponic model (24.4 [SD, 10.3 ]). There was an association between thoracic spine classification and cervical pain, with a higher prevalence of thoracic kyphosis and cervical pain in the traditional model and a higher prevalence of normal curvature in the hydroponic model. Both groups reported a higher prevalence of pain in the lower back than in other locations.

**Conclusions:**

The cultivation model influenced posture and back pain prevalence in strawberry producers. Producers who use the traditional model have greater angulations of the thoracic spine, hyperkyphosis, lumbar straightening, and cervical pain than those who use the hydroponic model.

## Abstract (pt)

**Introdução:**

O cultivo de morango é uma importante fonte de renda de agricultores brasileiros. Atualmente, o cultivo pode ser feito no modelo tradicional, em que o produtor flexiona o tronco para manusear a muda, ou no modelo hidropônico, em que ele adota uma postura ereta.

**Objetivos:**

Verificar a influência do modelo de cultivo na postura e na prevalência de dor na coluna vertebral em produtores de morango.

**Métodos:**

Participaram 26 produtores de morango de diferentes modelos de cultivo (tradicional e hidropônico). Os valores angulares das curvaturas da coluna torácica e lombar no plano sagital foram obtidos por meio do método “flexicurva”, e a prevalência de dor foi medida através do questionário Informações sobre Dor nas Costas. Os modelos foram comparados com o teste *t* para amostras independentes, e a associação, com o teste de qui-quadrado.

**Resultados:**

Os produtores de morango que plantam a fruta no modelo tradicional apresentam maiores curvaturas na coluna torácica (45,5 ± 26,2 ) quando comparados aos que utilizam o modelo hidropônico (24,4 ± 10,3 ). Houve associação entre as classificações da coluna torácica e dor cervical, demonstrando maior prevalência de hipercifose torácica e dor na cervical no modelo tradicional e curvatura normal no hidropônico. Ambos os grupos apresentam maior prevalência de dor lombar.

**Conclusões:**

Pode-se concluir que há influência do modelo de cultivo na postura e na prevalência de dor na coluna vertebral em produtores de morango. Os produtores que manuseiam a fruta no modelo tradicional possuem maiores angulações na coluna torácica, prevalência de hipercifose, retificação lombar e dores na região cervical em comparação aos que manuseiam a fruta no modelo hidropônico.

## INTRODUCTION

Strawberry (*Fragaria x ananassa*) is cultivated in the most varied regions of the planet,^1^ with increasing production every year.^2^ The fruit is an important source of income for many Brazilian farmers,^3^ especially small producers,^4^ having great value commercially and for the rural economy.^2^

**Figure 1:**
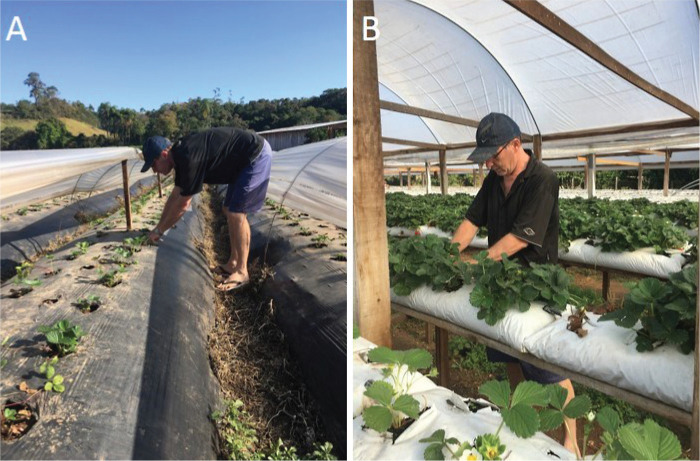
Strawberry cultivation methods. A = traditional cultivation; B = hydroponic cultivation.

Fruit cultivation requires dedication and skill,^2^ and in southern Brazil the most common cultivation models are the so-called conventional(traditional),^5^ hydroponic, and semi-hydroponic.^3^ The traditional cultivation model (Figure 1A) is carried out in the soil, in small plots of land, while the hydroponic model (Figure 1B) involves troughs at least 1 meter high.^3^

From an ergonomic point of view, the traditional model requires producers to flex their trunk to handle seedlings, while, in hydroponic cultivation, producers work in an upright posture. The literature reports that, due to the required posture, perceived pain and discomfort are greater in traditional model than in the more upright position used in the hydroponic model.^3^ A high prevalence of low back pain is also reported by rural workers.^6^ Farmers report that the highest rates of pain occur in activities such as preparing the soil and planting, treating, and harvesting crops, which are part of daily life for strawberry producers.^7^ Several other risk factors are involved, such as overexertion, repetitive movement, incorrect postures maintained for long periods, no work breaks, and a fast pace, which can lead to the emergence of musculoskeletal disorders.^8^

Although the literature has investigated pain perception among producers, larger samples are still needed to compare cultivation models and verify their effects on spinal curvature, due to the positions required in different work routines. From this perspective, this study aimed to verify the influence of the cultivation model on posture and the prevalence of back pain among strawberry producers.

## METHODS

### STUDY DESIGN

This quantitative study, which involved a descriptive, comparative and cross-sectional design,^9^ was approved by the Universidade do Vale do Rio dos Sinos Research Ethics Committee (protocol 4.312.062). The study was also authorized by the Bom Principe Association of Strawberry Producers. All participants provided written informed consent prior to inclusion.

### SAMPLE

A total of 26 strawberry producers from Bom Princípio, state of Rio Grande do Sul, Brazil, participated in the study. The participants used either traditional (41.7 ± 9.7 years; n = 13) or hydroponic (39.1 ± 8.1 years; n = 13) cultivation systems (Figure 1). Prospective participants were visited individually at home based on information provided by the Bom Principle Association of Strawberry Producers. Participants were selected by convenience, with the sample being intentional and not probabilistic. The eligibility criteria were: (1) working in only one type of crop; (2) having worked for at least 5 years with this crop type; and (3) no history of accidents or spinal surgeries.

### PROCEDURES

Data collection consisted of an initial interview regarding eligibility criteria and sociodemographic data, followed by pain and postural assessments. The evaluation was performed by an individual researcher according to the participant’s convenience.

Pain assessment was performed using Souza & Krieger’s^10^ Instrumento de Avaliação da Dor nas Costas (back pain assessment instrument), whose objective is to identify the intensity and frequency of pain in seven regions: the cervical spine, the shoulders, the shoulders and arm, the dorsal spine, the lower back, the gluteus muscles, and the gluteus muscles and legs.

The questionnaire consists of 2 parts. The first part presents the instrument’s objectives, identifies individual data and maps the human body, indicating the 7 above-mentioned regions. The second part investigates pain intensity in these regions according to the following scale: none, mild, average, strong, and unbearable. Pain frequency is assessed with the following alternatives: 1-4 times per year, 1-4 times per month, 1-4 times per week, 4-6 times per week, and 7 times per week. All those who answered “none” were considered pain-free; for any other option, the producer was classified as having pain.

**Figure 2:**
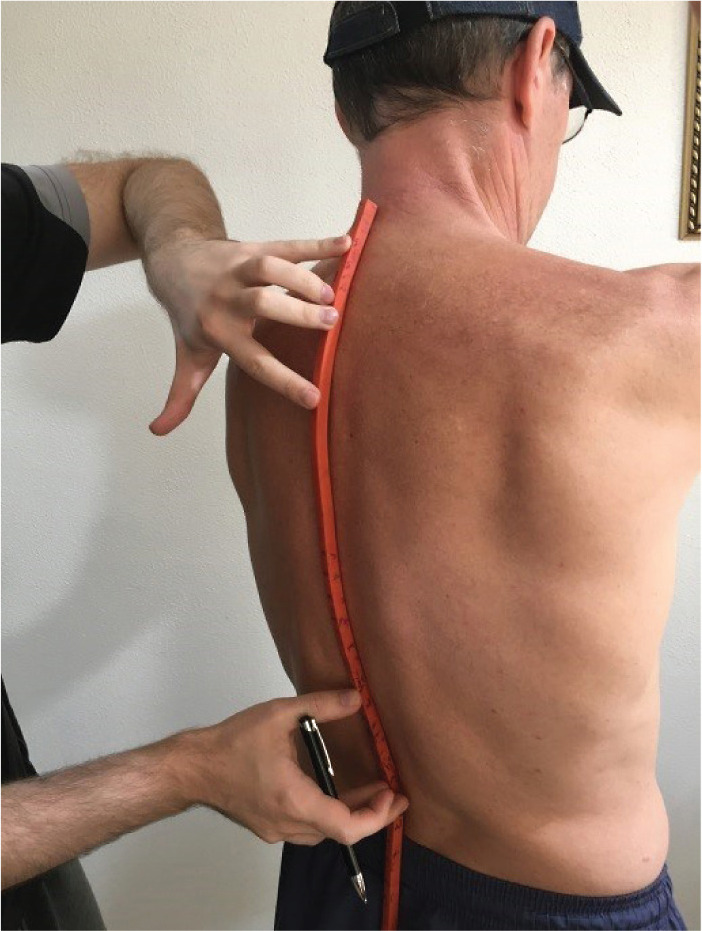
Postural assessment using the flexicurve method.

After the pain assessment was completed, postural assessment was performed using the “flexicurve” method, in which a flexible plastic-coated metal ruler is molded to the spinal curvature in the dorsal region and then traced on graph paper.^11,12^ To do this, participants stood in their usual posture with their back unclothed, their bare feet parallel, and their shoulders and elbows at 90° of flexion. To determine the thoracic curvature, the spinous processes of six anatomical points (C7, T1, T12, L1, L5 and S1) were located through palpation and marked with a skin pencil. After marking the anatomical points of interest, the flexible ruler was molded to the participant’s back from the C7 spinous process to the S1 spinous process (Figure 2). The points of interest were then marked on the ruler, it was removed from the participant’s back, and its internal contour (ie, in contact with the skin) was traced on A1 graph paper. This drawing represented the thoracic and lumbar sagittal curvatures, and the spinous processes of interest (C7, T1, T12, L1, L5 and S1) were also identified.

In each drawing, six random points were marked between C7 and T12 and between L1 and L5, totaling 18 points along the curvature. A digital image was then made of this outline.

### DATA ANALYSIS

The data were analyzed using BIOMEC FLEX 3.0, in which the 18 marked points were scanned and the spinal angles estimated by trigonometry, generating a graph and a report on the individual’s posture and thoracic and lumbar curvature (in degrees).

Angular values > 40° were considered hyperkyphotic posture, values < 20° were classified straightened curvature, and angles between 20° and 40° were considered intact physiological curvature.^13,14^ For the lumbar curvature, angles > 54° were considered hyperlordosis, angles < 22° were considered straightened curvature, and angles between 22° and 54° were considered intact physiological curvature.^14,15^

### STATISTICAL ANALYSIS

Statistical analysis was performed in IBM SPSS Statistics 20.0, including Student’s *t-*test for independent samples to compare the angle (mean and standard deviation) of lumbar and thoracic curvature between cultivation models. The chi-square test was used to assess the associations between frequencies (n and %) of pain (cervical, thoracic, and lumbar), postural deviations (hyperkyphosis, hyperlordosis, straightening, or none) and cultivation model. The significance level was set at 5%.

## RESULTS

The thoracic curvature of traditional producers was significantly greater than that of hydroponic producers (t = 2.702; p = 0.012). There was no difference between cultivation models regarding lumbar curvature (t = 1.268; p = 0.012) (Table 1).

**Table 1:** Mean and standard deviation of the thoracic and lumbar curvature of traditional and hydroponic strawberry producers

Variables	Traditional (n = 13)	Hydroponic (n = 13)	p-value
Thoracic spine curvature	45.5 ± 26.2	24.4 ± 10.3	0.012^*^
Lumbar spine curvature	23.8 ± 27.1	34.7 ± 15.2	0.217

* Significant difference (p < 0.05).

There was an association between thoracic (chi-square = 7.500; p = 0.024) and lumbar spine (chi-square = 6.623; p = 0.036) classifications and cultivation model, ie, a higher prevalence of hyperkyphosis and lumbar straightening in traditional cultivation, and a higher prevalence of normal curvature in hydroponic cultivation (Table 2). There was an association between cervical spine pain and cultivation model (chi-square = 6,190; p = 0.013), given that none of the hydroponic producers reported pain in this region. There was no association between thoracic spine pain (chi-square = 1,182; p = 0.277), lumbar spine pain, and cultivation model (chi-square = 1,182; p = 0.277). There was no association between thoracic pain, lumbar pain, and cultivation model, and the highest prevalence of pain was identified in this region.

**Table 2:** Pain frequency values and spinal curvature classification of traditional and hydroponic strawberry producers

	Traditional (n/%) n = 13	Hydroponic (n/%) n = 13	p-value
Thoracic curve			
Hyperkyphosis	7 (53.8)	1 (7.7)	
Normal	5 (38.5)	7 (53.8)	0.024^*^
Straightened	1 (7.7)	5 (38.5)	
Lumbar curve			
Hyperlordosis	3 (23.1)	2 (15.4)	
Normal	1 (7.7)	7 (53.8)	0.036
Straightened	9 (69.2)	4 (30.8)	
Thoracic pain			
Yes	3 (23.1)	1 (7.7)	
No	10 (76.9)	12 (92.3)	0.277
Lumbar pain			
Yes	12 (92.3)	10 (76.9)	
No	1 (7.7)	3 (23.1)	0.277
Cervical pain			
Yes	5 (38.5)	0 (0.0)	
No	8 (61.5)	13 (100.0)	0.013^*^

* Significant difference (p < 0.05).

## DISCUSSION

This study aimed to verify the influence of cultivation model on posture and back pain among strawberry producers. Those using the traditional model had greater thoracic curvature and a higher prevalence of thoracic hyperkyphosis, lumbar straightening, and cervical pain than hydroponic producers. However, growers from both models had a higher prevalence of pain in the lower back than the other regions.

These results may be related to the posture required for the traditional cultivation model, in which the fruit is grown in the ground. This model requires trunk flexion (Figure 1), the kinematic consequences^16^ of which are reduced lumbar lordosis (straightening) and thoracic hyperkyphosis, precisely what we found in the present study (Tables 1 and 2). Moreover, when growers have reduced hip flexibility, greater demand is placed on the lumbar spine to flex the trunk, which can increase overload in this region.^17^ This could explain why the highest prevalence of pain occurred in the lumbar region, regardless of cultivation model.

The literature reports that greater pain is associated with the traditional model than the semi-hydroponic model, which involves an upright position due to the use of troughs.^3^ Cervical, thoracic, and lumbar/sacral pain has also been reported in other farming activities, such as preparing the soil, planting, treating and harvesting crops.^7^ Lumbar and shoulder pain has also been reported by poultry workers due to the trunk flexion required to collect eggs and to clean chicks and drinkers.^18^ Other farming activities associated with a high prevalence of shoulder and low back pain are hoeing and spraying.^8^ Locksmithing, which involves similar trunk flexion to the traditional strawberry cultivation model, has also been associated with complaints of back pain.^19^ Another work activity involving spine flexion is dentistry. Although seated, these professionals must perform anterior trunk flexion daily to assist their patients, which can result in scoliosis, hyperkyphosis, and lumbar pain.^20^ In view of the above, our results agree with the literature, since the highest prevalence of pain among strawberry producers was in the lumbar region, regardless of the cultivation model.

Although the literature also reports higher pain rates in the lumbar region among strawberry producers, we found no difference in prevalence between cultivation models. However, there was an association between cervical spine pain and cultivation model. It is noteworthy that no hydroponic producer in our sample complained of cervical pain. This is probably due to the fact that in the traditional method spine flexion is accompanied by hyperextension of the cervical spine and the constant action of the head and neck extensors to maintain the posture (Figure 1). This posture generates greater flexor torque because, with the vertebral column inclined forward, the cervical curvature must be extended for the farmer to view the seedlings, which causes greater activation of the posterior head and neck muscles. Such effort by the erector spinae muscles for long hours can lead to fatigue, muscle imbalance, pain, and overload in the spine and intervertebral discs.^21^

This study was limited by not assessing cervical spine curvature or its association with cultivation model. The flexicurve method still lacks validation for cervical spine values. Thus, future studies can investigate the relationship between cervical curvature and pain in the traditional agricultural model. Another limitation was the decision to only evaluate producers who worked with a single model. Although we did this specifically to contrast any curvature and pain differences, future research may benefit from evaluating mixed production types. Moreover, we limited our sample to a single city in southern Brazil, which despite its fairly recent establishment and small size (14,055 inhabitants), is considered the strawberry capital of the country.

Our results are particularly relevant for professionals who deal directly with the spine and human movement (doctors, physical therapists, chiropractors, etc.), since they provide evidence of body posture and pain differences in different cultivation models. Based on these results, professionals can instruct producers to adapt their seedling management processes to prevent or control pain and the overloading associated with the traditional model. To our knowledge, our study is the first to evaluate spinal curvature among strawberry producers using the flexicurve method, which can be easily applied in clinical practice.

## CONCLUSIONS

It can be concluded that the cultivation model influences the posture and back pain prevalence of strawberry producers. Workers who use the traditional model have higher thoracic spinal angles and a higher prevalence of hyperkyphosis, straightening of the lumbar spine, and cervical pain than those who use the hydroponic model. However, in both models the highest prevalence of pain was in the lower back.
